# Supplementary material for: Charting the native architecture of Chlamydomonas thylakoid membranes with single-molecule precision
Source: eLife. 2020 Apr 16;9:e53740. doi: 10.7554/eLife.53740 (PMC7164959; doi:10.7554/eLife.53740)
Supplement: Transparent reporting form [file elife-53740-transrepform.docx]

***eLife’s* transparent reporting form**

We encourage authors to provide detailed information *within their submission* to facilitate the interpretation and replication of experiments. Authors can upload supporting documentation to indicate the use of appropriate reporting guidelines for health-related research (see [EQUATOR Network](http://www.equator-network.org/%20)), life science research (see the [BioSharing Information Resource](https://biosharing.org/)), or the [ARRIVE guidelines](http://www.plosbiology.org/article/info:doi/10.1371/journal.pbio.1000412) for reporting work involving animal research. Where applicable, authors should refer to any relevant reporting standards documents in this form.

If you have any questions, please consult our Journal Policies and/or contact us: [editorial@elifesciences.org](mailto:editorial@elifesciences.org).

**Sample-size estimation**

- You should state whether an appropriate sample size was computed when the study was being designed
- You should state the statistical method of sample size computation and any required assumptions
- If no explicit power analysis was used, you should describe how you decided what sample (replicate) size (number) to use

Please outline where this information can be found within the submission (e.g., sections or figure legends), or explain why this information doesn’t apply to your submission:

No explicit power analysis was used. The sample size was limited by the technical constraints of high-quality Volta phase plate cryo-ET imaging, as stated in the methods section:

**Methods: Tomogram Reconstruction**

Of the 13 Volta phase plate tomograms acquired, four tomograms (Figs. 1 and S1) were selected for analysis of photosynthetic complexes based on good IMOD tilt-series alignment scores and visual confirmation of well-resolved complexes at the thylakoid membranes.

For transparency, all four tomograms and the segmented membranes that were quantified are displayed in **Figures 1 and S1**.

This data set allowed quantification of 4082 protein densities within 84 membrane regions.

**Replicates**

- You should report how often each experiment was performed
- You should include a definition of biological versus technical replication
- The data obtained should be provided and sufficient information should be provided to indicate the number of independent biological and/or technical replicates
- If you encountered any outliers, you should describe how these were handled
- Criteria for exclusion/inclusion of data should be clearly stated
- High-throughput sequence data should be uploaded before submission, with a private link for reviewers provided (these are available from both GEO and ArrayExpress)

Please outline where this information can be found within the submission (e.g., sections or figure legends), or explain why this information doesn’t apply to your submission:

**Methods: 77K Measurements**

The average 77K profile for each condition was composed of three independent biological replicates, each with five technical replicates (15 measurements total per condition).

**Methods: Cryo-ET**

Each tomogram was acquired from a separate cell and thus is both a biological and technical replicate.

**Methods: Tomogram Reconstruction**

Of the 13 Volta phase plate tomograms acquired, four tomograms (Figs. 1 and S1) were selected for analysis of photosynthetic complexes based on good IMOD tilt-series alignment scores and visual confirmation of well-resolved complexes at the thylakoid membranes.

**Statistical reporting**

- Statistical analysis methods should be described and justified
- Raw data should be presented in figures whenever informative to do so (typically when N per group is less than 10)
- For each experiment, you should identify the statistical tests used, exact values of N, definitions of center, methods of multiple test correction, and dispersion and precision measures (e.g., mean, median, SD, SEM, confidence intervals; and, for the major substantive results, a measure of effect size (e.g., Pearson's r, Cohen's d)
- Report exact p-values wherever possible alongside the summary statistics and 95% confidence intervals. These should be reported for all key questions and not only when the p-value is less than 0.05.

Please outline where this information can be found within the submission (e.g., sections or figure legends), or explain why this information doesn’t apply to your submission:

**Figures 4, S4, and S6** are the only figures with statistical measurements. In Fig. 4, the figure legend lists N values and states the that the error bars are standard deviation. In Figs. S4 and S6, the Mean, Median, and SD are specified on the figure.

**Table 1**, which lists the concentrations of thylakoid-bound complexes, also states:

N= 84 membrane regions (51 non-appressed and 33 appressed) from four tomograms.

**Figure 4** is the only figure with statistical tests. Exact p-values are listed, e.g.:

The experimental measurements (PSII: 8.0% ± 0.6%, LHCII: 5.8% ± 0.7%) and simulations of complexes randomly positioned within the membrane (PSII: 7.5% ± 0.6%, LHCII: 6.4% ± 0.6%, N= 100 simulations per overlay) were not significantly different (P > 0.05 from t-test with Welch’s correction for unequal variance: P= 0.174 for PSII, P= 0.197 for LHCII).

(For large datasets, or papers with a very large number of statistical tests, you may upload a single table file with tests, Ns, etc., with reference to sections in the manuscript.)

**Group allocation**

- Indicate how samples were allocated into experimental groups (in the case of clinical studies, please specify allocation to treatment method); if randomization was used, please also state if restricted randomization was applied
- Indicate if masking was used during group allocation, data collection and/or data analysis

Please outline where this information can be found within the submission (e.g., sections or figure legends), or explain why this information doesn’t apply to your submission:

No experimental groups were used in this study.

**Additional data files (“source data”)**

- We encourage you to upload relevant additional data files, such as numerical data that are represented as a graph in a figure, or as a summary table
- Where provided, these should be in the most useful format, and they can be uploaded as “Source data” files linked to a main figure or table
- Include model definition files including the full list of parameters used
- Include code used for data analysis (e.g., R, MatLab)
- Avoid stating that data files are “available upon request”

Please indicate the figures or tables for which source data files have been provided:

Tomograms will be made available on the Electron Microscopy Data Bank (EMDB).

The membranorama software is freely available at: <https://github.com/dtegunov/membranorama>
